# Global antimicrobial resistance—The ostrich’s head is in the sand

**DOI:** 10.1371/journal.pbio.3003382

**Published:** 2025-09-08

**Authors:** Susanna J. Dunachie, Mariagrazia Pizza

**Affiliations:** 1 NDM Centre for Global Health Research, Nuffield Department of Medicine, University of Oxford, Oxford, United Kingdom; 2 Mahidol-Oxford Tropical Medicine Research Unit, Mahidol University, Bangkok, Thailand; 3 NIHR Oxford Biomedical Research Centre, Oxford University Hospitals NHS Foundation Trust, Oxford, United Kingdom; 4 Centre for Bacterial Resistance Biology (CBRB), Imperial College London, London, United Kingdom

## Abstract

Antimicrobial resistance (AMR) is a growing problem, ignored at our peril. This Perspective outlines the technical difficulties and challenges raised by the lack of market incentives for new diagnostics, drugs and vaccines to tackle AMR, and discusses potential solutions.

## Teaser

Antimicrobial resistance (AMR) is a growing problem that should be ignored at our peril. The development of new diagnostics, drugs, and vaccines to tackle AMR faces technical difficulties and challenges that need to be addressed urgently.

Antimicrobial resistance (AMR) is a huge and growing problem. Estimates of the global burden of AMR are startling, with nearly 5 million deaths worldwide associated with AMR in 2019 [1]: almost double the estimate of deaths that year for malaria, HIV, and tuberculosis combined [2]. Sadly, AMR may not get the attention it needs until we reach a world with widespread untreatable infections, where people in high-income countries cannot undergo surgery or immunosuppressive treatments due to the overwhelming risks of incurable infections. Global “duty bearers” in government, international organizations, and the private sector, alongside society in general, are hiding their heads in the sand and avoiding the issue. Change is needed before the current under-investment in solutions becomes dangerous in the next few years.

Novel antibacterial agents are being developed, with 57 traditional antibacterial agents and 40 nontraditional agents (bacteriophages, phage-derived enzymes, antibodies, etc.) listed in the clinical antibacterial pipeline in 2023 [3]. However, the current pipeline is insufficient to keep up with the emergence and spread of AMR, and attention to therapeutic development for babies and children is lacking, particularly in light of the scale of AMR-related neonatal deaths in low-resource settings [4]. Development of novel antimicrobial agents is challenging due to the difficulties of finding effective yet nontoxic drugs, and the specter of rapidly emerging resistance to new agents. Incentives for commercial research and development (R&D) for AMR drug discovery are poor due to pharmaceutical companies perceiving the area as being ‘high risk without high gain’. Antimicrobial stewardship measures are likely to restrict the use of successful newly launched antimicrobial agents to preserve their effectiveness, and low volume-based sales are an unattractive commercial prospect. Above all, R&D is not targeting interventions that are relevant to the Global South, where most of the consequences of AMR reside. Instead, the focus remains on profitable products for high-income countries, using a drug development framework that has failed to adapt to modern needs. We cannot leave free market capitalism to solve AMR for this reason; it is a ‘wicked problem’, akin to climate change or drug trafficking, that will require international cooperation and treaties to tackle.

We need better economic incentives to stimulate the scale of ongoing R&D, which is required for progress. A number of countries have explored a subscription-based model, in which governments or healthcare entities pay a fixed annual subscription fee to a pharmaceutical company in return for access to new antibiotics when needed [5]. In the UK, the NHS Antimicrobial Products Subscription Model, colloquially referred to as the “Netflix model”, has awarded contracts to Pfizer and Shionogi for antibiotics to treat drug-resistant gram-negative bacteria. Other regions have piloted payment models, including the EU’s Health Emergency Preparedness and Response Authority, which is testing various EU-wide ‘pull-incentive’ models, but these have yet to generate the momentum we need.

One future option is mandatory twinning of the development of novel immunosuppressants or chemotherapies with matched R&D or levy payments for new antimicrobials to treat the infections that occur with the use of such therapies. Another option under discussion is coupling drug development with scalable bedside diagnostic tests for AMR, although the relationship between a detectable resistance mechanism and the optimal therapeutic solution is not always a simple match, as patient factors, infection features, and bacterial traits contribute to tailored antibiotic choices in clinical practice. Bacteriophage therapy is another alternative or adjuvant to antibiotics but requires further attention and investment; WHO is leading a dialogue on building the evidence base for bacteriophage therapy in collaboration with the Global Antimicrobial Resistance R&D Hub [6].

Vaccines remain an outstanding public health intervention to prevent and control infection [7,8]. However, there is scope to use existing vaccines better, both against bacteria showing AMR (e.g., *Streptococcus pneumoniae*, *Salmonella* Typhi), and against viruses (e.g., influenza, respiratory syncytial virus, SARS-CoV-2) to reduce the impact of inappropriate antimicrobial prescribing and risk of secondary bacterial infections following viral illnesses. We also need new and improved vaccines to prevent infection from major pathogens showing AMR, such as **Mycobacterium tuberculosis*, *Klebsiella pneumoniae*,* and *Neisseria gonorrhoeae*. Many candidates are under development in the academic and commercial sector, with some interesting approaches including whole-cell vaccines, synthetic polysaccharide and glycoconjugates for Shigella and outer membrane vesicle vaccines also for a range of bacteria, such as *N. gonorrhoeae* and *Acinetobacter baumannii* [8]. The disappointing result of the phase III clinical trials of the Johnson & Johnson vaccine candidate for extraintestinal pathogenic *Escherichia coli* [9] was therefore very sobering news for anti-bacterial vaccines. This candidate was based on the polysaccharide O antigen and induced high levels of functional antibodies, but the clinical trial did not reproduce the protection seen against invasive disease in animal models.

Yet, rather than giving up on vaccines as an approach to AMR control, we instead need to undertake a deeper understanding of human immunity to bacteria in health and disease and identify potential correlates of protection; this includes understanding the role of T cells, which are a foundation of defense against many infections. Parallel work can also enable development of effective monoclonal antibody cocktails for prophylaxis and treatment. In addition, there is potential to design vaccines against key resistance mechanisms to restore antimicrobial susceptibility. However, keeping up with the acquisition and spread of resistance genes, including by plasmids, is immensely challenging. Opportunities also exist to develop multi-valent anti-disease vaccines that target key virulence factors, thereby reducing disease severity and death following infection. This approach is especially important for bacteria such as *E. coli* that form a key part of our host flora and are ubiquitous in agriculture and animals. Bacterial elimination by vaccination is neither feasible nor desirable in these cases, given the importance of the gut microbiome in regulating our immune systems and the fact that these bacteria occupy niches that could instead be held by pathogens of higher virulence.

While excess antibiotic use is a global problem, for much of the Global South, access to appropriate antibiotics is the issue, with a lack of planning, finance, and infrastructure to deliver existing drugs to the patients who need them most. Antibiotics are therefore in the wrong places. A crucial knowledge gap is how our approach to the diagnosis of infection should adapt across differing resource-level settings to better support the proper use of antibiotics. As such, we welcome the newly launched global AMR diagnostics collaboration (DxAMR), which brings together key stakeholders including the Fleming Initiative, GARDP, and FIND, alongside WHO, funders, and international governments. More basic science will be essential to identify and develop new diagnostic tools that are scalable and affordable, alongside pipelines to evaluate and deploy existing tools and new candidates. Infection prevention and control strategies are also important, including isolation of patients colonized or infected with AMR pathogens.

Moving forward, public engagement around the use of antibiotics will be crucial to learn others’ perspectives and share concerns. The risks of AMR to human health sit within the wider One Health ecosystem, which includes the environment, agriculture, and farming. Listening to and funding these research communities will be vital for a joined-up and secure approach to AMR. Modeling and health economic analysis are also important to understand the impact of different interventions and guide policy decisions. The world needs global infrastructure for pandemic preparedness for diagnostic testing and manufacture of drugs and vaccines, and with political and financial prioritization, we could establish and maintain this infrastructure in ‘peacetime’ between pandemics to tackle AMR.

Ultimately, AMR is a global problem that will require international cooperation and treaties to address and to ensure concerns translate into funding investment for the manufacture of new solutions, be they drugs, vaccines, monoclonal antibodies, bacteriophages, or diagnostics. As such, huge cuts in global funding for aid in 2025 by the US and UK governments are a disaster for AMR, including the recent UK government announcement to close the Fleming Fund for AMR. We need people to wake up and realize the dangers of a future with untreatable infections.

## Abbreviations

AMR: antimicrobial resistance
R&D: research and development
